# Galectin-3, Possible Role in Pathogenesis of Periodontal Diseases and Potential Therapeutic Target

**DOI:** 10.3389/fphar.2021.638258

**Published:** 2021-03-19

**Authors:** Milica Velickovic, Aleksandar Arsenijevic, Aleksandar Acovic, Dragana Arsenijevic, Jelena Milovanovic, Jelena Dimitrijevic, Zeljko Todorovic, Marija Milovanovic, Tatjana Kanjevac, Nebojsa Arsenijevic

**Affiliations:** ^1^ Department of Dentistry, Faculty of Medical Sciences, University of Kragujevac, Kragujevac, Serbia; ^2^ Center for Molecular Medicine and Stem Cell Research, Faculty of Medical Sciences, University of Kragujevac, Kragujevac, Serbia; ^3^ Department of Pharmacy, Faculty of Medical Sciences, University of Kragujevac, Kragujevac, Serbia; ^4^ Department of Histology and Embriology, Faculty of Medical Sciences, University of Kragujevac, Kragujevac, Serbia; ^5^ Department of Internal Medicine, Faculty of Medical Sciences, University of Kragujevac, Kragujevac, Serbia

**Keywords:** periodontal diseases, galectin-3, gingival barrier, periodontal immunity, dysbiosis

## Abstract

Periodontal diseases are chronic inflammatory diseases that occur due to the imbalance between microbial communities in the oral cavity and the immune response of the host that lead to destruction of tooth supporting structures and finally to alveolar bone loss. Galectin-3 is a β-galactoside-binding lectin with important roles in numerous biological processes. By direct binding to microbes and modulation of their clearence, Galectin-3 can affect the composition of microbial community in the oral cavity. Galectin-3 also modulates the function of many immune cells in the gingiva and gingival sulcus and thus can affect immune homeostasis. Few clinical studies demonstrated increased expression of Galectin-3 in different forms of periodontal diseases. Therefore, the objective of this mini review is to discuss the possible effects of Galectin-3 on the process of immune homeostasis and the balance between oral microbial community and host response and to provide insights into the potential therapeutic targeting of Gal-3 in periodontal disease.

## Introduction

Periodontal diseases include a number of inflammatory conditions that affect the supporting structures of the teeth, gingiva, bone and periodontal ligament (Kinane et al., 2017). Periodontitis is an inflammatory disease of periodontal tissue characterized by activation of host-derived proteinases that mediate loss of tooth supporting tissue (Tonetti et al., 2018). According to the newest classification, the forms of the disease previously recognized as “chronic” and “aggressive” are now grouped under a single category, periodontitis (Chapple et al., 2018; Papapanou et al., 2018). Periodontal diseases arise when the balance between the microbiota and the host is lost, due to the dysbiosis in the dental plaque microbiota (Darveau, 2010; Costalonga and Herzberg, 2014; Yost et al., 2015) or by changes of the host inflammatory/immune reaction to microbes (Kinane et al., 2007; Benakanakere and Kinane, 2012; Hajishengallis and Lamont, 2012; Feres et al., 2015). The result of the disturbed homeostasis is a boosted inflammation that finally causes damage of the periodontal tissue (Graves, 2008), including ressorption of alveolar bone by osteoclasts, degradation of ligaments by matrix metalloproteinases and the formation of granulation tissue (Gemmell and Seymour, 2004; Sorsa et al., 2016).

Early studies indicated that periodontitis can be initiated with a single microbe, *Bacteroides gingivalis* (Holt et al., 1988). Later studies reported that periodontitis severity is associated with specific red clusters of microbes (*Porphyromonas gingivalis, Tannerella forsythia*, *Aggregatibacter actinomycetemcomitans,* and *Treponema denticola*) in oral cavity, while the green complex with dominant *Streptococcus* species, is associated with periodontal health (Socransky et al., 1998; Periasamy and Kolenbrander, 2009; Haubek, 2010; Kolenbrander et al., 2010; Pérez-Chaparro et al., 2014; Pérez-Chaparro et al., 2016). Newer theory, that takes into account the fact that periodontal pathogens are often low abundant in periodontits and can be present in people with periodontal health (Haffajee et al., 1998), indicates that the key pathogens change the composition of the microbiota thereby generating modulation of immune response, dysbiosis, tiisue hoemostasis disruption, leading to the periodontitis (Hajishengallis et al., 2011; Hajishengallis and Lamont, 2012). Now, the well-known fact is that the dysbiosis in oral microbiota is the pathogenic (Feres et al., 2016) and periodontitis is initiated and propagated by the activity of the entire subgingival microbial community of affected sites, characterized by higher amounts of pathogens, and decreased proportion of commensal microorganisms (Faveri et al., 2009; van Essche et al., 2013) and not by a single pathogen (Fine et al., 2013), and its interaction with immune system of the host (Darveau, 2010). The intensity of the host inflammatory response to microbial plaque is an invidual feature affected by the host genetic factors (Tatakis and Trombelli, 2004). The polimorfism of IL-1RN is associated with clinical manifestations in the gingiva in response to *de novo* plaque formation (Scapoli et al., 2005). Higher levels inflammatory mediators, tumor necrosis factor-α (TNF-α), IL-1 and prostaglandin E2, correlate with the extent of periodontal damage (Stashenko, 1990).

Galectin-3 (Gal-3) is an evolutionarily conserved lectin produced by various cell types including immune and epithelial cells and fibroblasts that affects the function of immune cells and has controversial pro- or anti-inflammatory activities depending on various factors including its intracellular or extracellular localization and the target cell implicated in the processes (Yang et al., 2008). Also, Gal-3 is a key component in the host defense against microbes, it interacts directly with different microbes playing the role of pathogen-associated molecular pattern (PAMP) receptor (Vasta, 2012), and also play the role of damage associated molecular pattern (DAMP) molecule (Sato et al., 2014). Gal-3 released from cells after microbial infections directly stimulates migration and production of inflammatory mediators in innate immune cells (Mishra et al., 2013; Sato et al., 2014). Although it is known that Gal-3 affects the key processes involved in the pathogenesis of periodontal diseases, inflammatory/immune responses and antimicrobial activity, there is no data about its possible role in development of these diseases, except few reports about Gal-3 expression in periapical lesions in humans (de Oliveira et al., 2014; Brito et al., 2018; Brito et al., 2018). The aim of this review is to, according to results obtained from investigation of Gal-3 impact on maintenance of epithelial cell, microbes, immune cells and its role in development of different inflammatory diseases, give a relevant rationale for a possible role of Gal-3 in pathogenesis of periodontal diseases.

## Structure and Functions of Galectin-3

**FIGURE 1:**
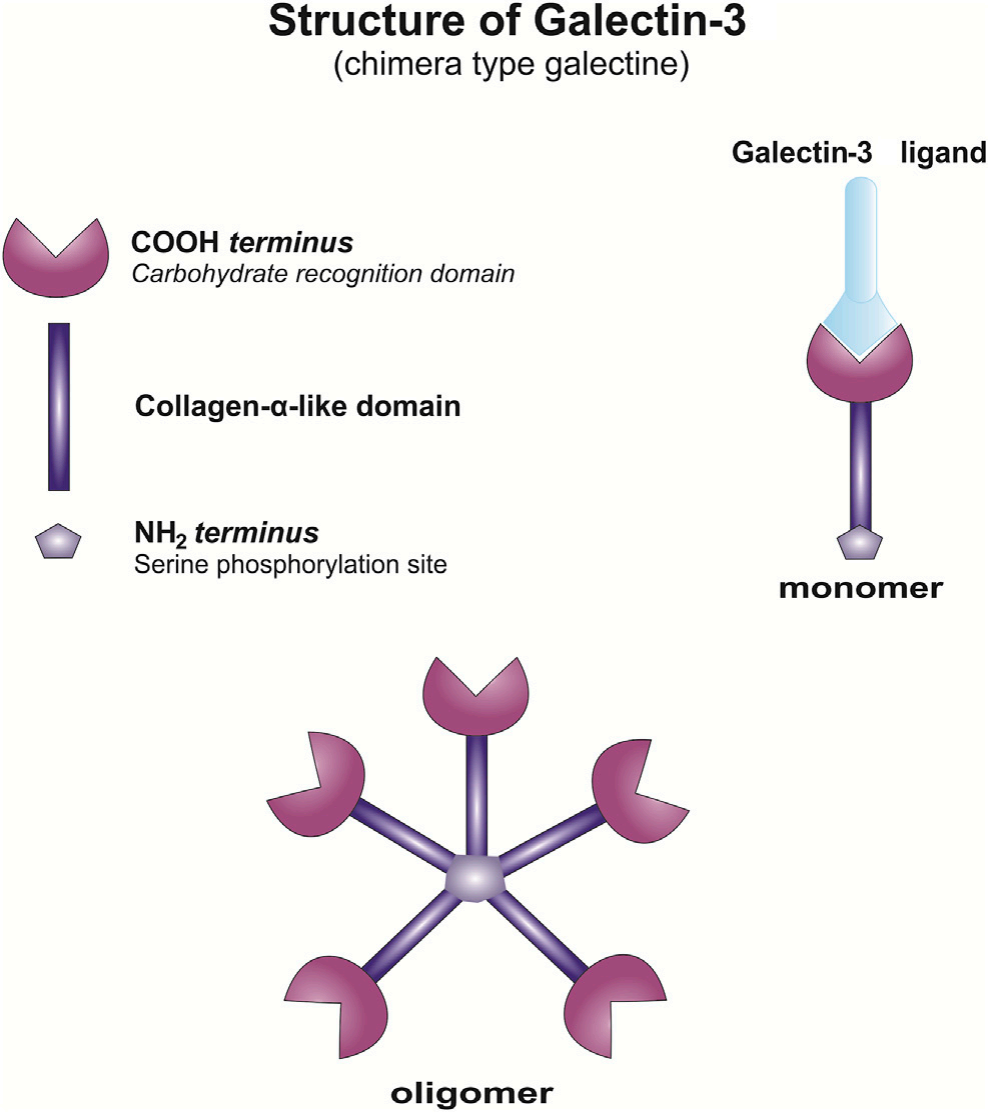
Structure of Gal-3. Schematic presentation of Gal-3 monomer and Gal-3 oligomerization.

Galectins are a family of evolutionary conserved proteins with carbohydrate-recognition domains (CRD) consisting of about 135 amino acids that make up the globular structure with high affinity for β-galactosides (Barondes et al., 1994; Leffler, 2001), gained an increased interest as therapeutic targets in chronic inflammatory diseases and cancer (Barondes et al., 1994; Liu and Rabinovich, 2010; Newlaczyl and Yu, 2011). There are 15 reported members of Galectin family classified into three groups 1) prototype, containing a single carbohydrate-recognition domain (CRD); 2) tandem repeats that harbor two CRDs and 3) chimera (Nangia-Makker et al., 2018). Galectin-3, the only one member of chimera type galectins, is structurally composed of a large N-terminal domain and one CRD (de Waard et al., 1976; Gong et al., 1999). Gal-3 contains three structurally distinct domains: 1) a short amino terminal consisting of 12 amino acids with a serine phosphorylation site responsible for its translocation (Gong et al., 1999) 2) a collagen-alpha like nearly 110 amino acid long structure rich in proline, alanine, glycine; and 3) C-terminal domain with nearly 140 amino acids encompassing the CRD (Figure 1). N-terminal tail of Gal-3 provides a stable structure to the whole molecule (Ochieng et al., 1998). Collagen-like domain of Gal-3 participates in the oligomerization of Gal-3 molecules and in its interactions with distinct proteins (Mehul et al., 1995; Ippel et al., 2016). Cleavage of collagen-like domain of Gal-3 by matrix metalloproteases (MMP), MMP-2 and MMP-9 (Ochieng et al., 1994; Nangia-Makker et al., 2008; Nangia-Makker et al., 2010) and prostate specific antigen (PSA) (Balan et al., 2012) enhances interactions of larger Gal-3 fragments with proteins leading to enhanced heterotypic aggregation, chemotaxis, and consequently tumor progression (Nangia-Makker et al., 2008; Nangia-Makker et al., 2010). The C-terminal CRD takes a form of β-sandwich with a tryptophan core and contains a noncanonical carbohydrate-binding site that allows interactions with sugars such as N-acetyllactosamine, galactomannans, and polymannan (Miller et al., 2016) and contains the characteristic NWGR anti-death motif of the bcl-2 family (Akahani et al., 1997). Galectins are proteins synthesized on free ribosomes in the cytosol, that although lack the typical characteristics of secretory proteins, rapidly translocates into the extracellular space and circulation *via* a nonclassical secretory pathways, *via* the exosome-mediated pathway or it may transverse the lipid bilayer (Menon and Hughes, 1999; Baptiste et al., 2007; Nakajima et al., 2016; Johannes et al., 2018).

In the extracellular environment, Gal-3 recognizes various glycan structures, is able to bind specific β-galactosides and to form multimers, to cross-link glycoconjugates and to form lattices (Figure 1). Agglutination of Gal-3 and aggregation with ligands occurs at low concentrations, while in physiological and high concentrations, Gal-3 is in the form of monomers (Massa et al., 1993; Lepur et al., 2012). This way of glycans latices organization on the cell surface enables the activation of signaling pathways in the cell (Ochieng et al., 2002). Gal-3 lattices present on the plasma membrane influence expression, localization, and activity of surface receptors and thus influence various biological functions ie. cell signaling, cell migration, cell adherence (Nabi et al., 2015; Johannes et al., 2018; Stegmayr et al., 2019). Further, galectins are also able to quickly recycle back to the cells trough the endocytosis (Furtak et al., 2001). Intracellular Gal-3 affects numerous functions, cell proliferation (Paz et al., 2001; Shalom-Feuerstein et al., 2008; Kaur et al., 2018), survival (Yang et al., 1996; Akahani et al., 1997), modulation of signaling activity (Bambouskova, 2016), regulation of mRNA splicing (Dagher et al., 1995; Park et al., 2001; Wang et al., 2004; Haudek et al., 2010). In intracellular space, galectins and also Gal-3, by interaction with proteins such as RAS proteins, β-catenin, CBP70, Chrp, Gemin4, Alix/AIP-1, and Bcl-2, affect cell signaling and by interaction with components of the nuclear spliceosome complex influence RNA splicing (Dagher et al., 1995; Akahani et al., 1997; Menon et al., 2000; Park et al., 2001; Liu et al., 2002; Elad-Sfadia et al., 2004; Shimura et al., 2004; Fritsch et al., 2016). However, it has been shown that several of these intracellular activities could be inhibited by molecules, such as lactose, that interacts with CRD of the galectin (Dagher et al., 1995; Shimura et al., 2004; Haudek et al., 2010). Gal-3 also play an important role at the interface between the cytosol and intravesicular spaces by controling the integrity of vesicular membranes. Gal-3 accumulates around damaged vesicles, binds exposed glycan structures and induce authophagy responsible for the clearance of the damaged organelles (Paz et al., 2010; Chauhan et al., 2016; Jia et al., 2020).

Gal-3 is constitutively expressed by myeloid cells (monocytes, macrophages, dendritic cells (DC), natural killer (NK) cells, mastocytes, neutrophils (Frigeri and Liu, 1992; Truong et al., 1993; Swarte et al., 1998; Sano et al., 2000; Dabelic et al., 2006; Tsuboi et al., 2011) and activated T and B lymphocytes (Acosta-Rodríguez et al., 2004; Peng et al., 2008). In adults it can be found in endothelial and epithelial cells, fibroblasts, chondrocytes and osteoblasts, osteoclasts, and epithelial cells of numerous organs (Iacobini et al., 2017; Simon et al., 2017). The various roles of Gal-3 on a cellular level are reflected on several physiological processes, in particular, related to immune responses and inflammation, as well as pathological conditions (Liu and Rabinovich, 2010; Newlaczyl and Yu, 2011; Sciacchitano et al., 2018). Extracellular Gal-3 may be present as a membrane associated or free, and participate in numerous processes such as the immune response against numerous pathogens, both acute and chronic inflammation, and apoptosis induction (Hsu and Liu, 2002; Hsu et al., 2006). Therefore Gal-3 can be viewed as a molecule with various, often opposite roles in acute inflammation and chronic inflammatory diseases depending on the stage of the inflammatory process.

## Possible Impact of Gal-3 on the Oral Tissue Homeostasis and the Periodontal Diseases Development

There are few data about Gal-3 expression in periodontal diseases. Significantly higher expression of Gal-3 in periapical granulomas and radicular cysts than in the control group has been reported, and also probably higher expression of Gal-3 in two examined granulomas than in cysts (de Oliveira et al., 2014). Results of other study conducted by Weber et al. show higher expression of Gal-3 in radicular cysts compared to apical granulomas and lower expression in dentigerous cysts, non inflammatory cause (de Moraes et al., 2014) developmental odontogenic cysts (Wright et al., 2014), than in both periapical pathologies (Weber et al., 2019), while Brito et al. reported no significant differences in Gal-3 immunoexpression in periapical granulomas, radicular cysts, and residual radicular cysts (Brito et al., 2018). Also, osteonecrosis of the jaw associated with bisphosphonates has been shown to be associated with significant increase of Gal-3 expression (Wehrhan et al., 2011).

## Epithelium and Gal-3

Epithelial healing involves coordinated migration and proliferation of epithelial cells, processes that are controlled by the activity of small GTP-ases (Yamaguchi et al., 2015). It is known that Gal-3 interacts with small GTP-ases activates kinases of Raf-1/MEK/ERK signaling pathway and induces proliferation of the cells (Shalom-Feuerstein et al., 2005). There are several reports about role of Gal-3 in re-epithelialization. Epithelial healing was the first time associated with Gal-3 when enhanced surface expression of Gal-3 was found in type I and II alveolar epithelial cells in a model of irradiation-induced lung inflammation at the time of active proliferation of type II alveolar epithelial cells and re-epithelialization of alveolar basement membranes by type I cells (Kasper and Hughes, 1996). Gal-3 promotes the migration of corneal epithelial front following corneal injury in mice and thus contributes to corneal re-epithelialization, and Gal-3 deficient mice exhibit impaired re-epithelialization (Cao et al., 2002). Later it has been shown that Gal-3 promotes cell scattering, lamellipodia formation, and motility in human corneal epithelial cells (Saravanan et al., 2009). Epithelial wounds healing in monkey corneal explants and enhanced re-epithelization are enhanced when the explants were mainteined in the presence of exogenous recombinant human Gal-3 (Fujii et al., 2015). It has been shown that skin re-epithelization also depends on Gal-3, since keratinocytes from Gal-3 deficient mice exhibit impaired migration (Liu et al., 2012) leading to delayed re-epithelialization (Liu et al., 2012; Walker et al., 2016). High expression of Gal-3 has been found in normal oral epithelial cells, significantly higher in comparison with epithelial cells in oral papiloma or squamous cell carcinoma (Hossaka et al., 2014). Production of Gal-3 in gingival epithelial cells can be rapidly stimulated *in vitro* with live and heat-killed *Candida albicans* and *Candida parapsilosis* (Tamai and Kiyoura, 2014). However there is no data about potential role of Gal-3 in maintenance of oral mucosal barrier. Since Gal-3 has the role in healing of corneal epithelium (Cao et al., 2002) and skin (Liu et al., 2012) it is possible that Gal-3 secreted in oral epithelium coud contribute to healing of junctional epithelium, constantly damaged by mastication and hygiene procedures. However, the role of Gal-3 in cell adhesion, assembly and stabiliazation of intercellular junctions could be dual, positive and negative and it depends on the level of expression and location of Gal-3 (Hughes, 2001). Gal-3 can be the critical factor that initiates cell–cell disassembly and promotes cell motility by inducing expression of MMP-9 (Mauris et al., 2014). This mechanism, by induction of cell motility, could be important for junctional epithelium healing in homeostasis, however, in disbiosis and presence of highly pathogenic microbes when Gal-3 could be overexpressed (Tamai and Kiyoura, 2014), Gal-3 could have opposite role and to contribute to tissue damage, due to the increased expression of MMP-9, proteinase with known role in periodontitis development (Checchi et al., 2020).

## Interactions of Immune System and Microbiota in Maintaining the Homeostasis at the Oral Mucosal Barrier and Gal-3

### Possible Interactions of Gal-3 and Microbiota

Microbiota plays the key role in the maintaining immune homeostasis in mucosal oral tissues. Oral microbial community is one of the most rich and diverse communities (Griffen et al., 2012), consisting of over 600 taxa, with the dominant *Firmicutes*, *Proteobacteria*, *Actinobacteria*, and *Bacteroidetes phyla* (Dewhirst et al., 2010), that form the complex, structurally organized, biofilm (Papapanou, 2012; Proctor and Relman, 2017). Dysbiosis of oral microbiota is the main trigger of a chronic inlammation in periodontal tissue resulting in damage of supporting structures and development of periodontal disease (Darveau, 2010; Hajishengallis and Hajishengallis, 2014).

Infection with different microbes increases Gal-3 expression, but underlying epigenetic mechanism that control this Gal-3 up-regulation remains poorly understood. It is known that different inflammatory stimuli activate Gal-3 transcription in the liver cells by direct binding of Brahma related gene (BRG1) to the Gal-3 promoter (Li et al., 2019). *Helicobacter pylori* enhances expression of Gal-3 in gastric epithelial cells (Lim et al., 2003; Fowler et al., 2006), *Neisseria meningitidis* induces Gal-3 expression in spleens in humans and mice (Quattroni et al., 2012), while *Candida albicans* and *Candida parapsilosis* upregulate the secretion of Gal-3 in human gingival epithelial cells (Tamai and Kiyoura, 2014) and neutrophils (Linden et al., 2013). Disbiosis of oral microbiota could be also the trigger for enhanced expression of Gal-3 in gingival epithelial cells, which could have dual roles, returning to homeostasis or further disruption the microbiota-immune system homeostasis.

Extracellular Gal-3 can act as the pattern recognition receptor for bacteria, virus, fungi, and parasites (Vasta, 2012; Chen et al., 2014). Gal-3 binds LPS from *Escherichia coli* (Fermino et al., 2011) and *Pseudomonas aeruginosa* (Gupta et al., 1997) resulting in oligomerization of Gal-3 and significantly potentiated proinflammatory activity of Gal-3 on neutrophils (Fermino et al., 2011). Also enhanced production of proinflammatory cytokines upon stimulation with LPS has been observed in Gal-3 deficient macrophages resulting in higher susceptibility of Gal-3 deficient mice to LPS-induced endotoxic shock (Fermino et al., 2011) but these mice showed increased resistance to infection by *Salmonella* spp. associated with a stronger Th1 immune response and higher production of superoxide anions (Li et al., 2008). Gal-3 has also been demonstrated to bind LPS from *Neisseria gonorrhoeae* (John et al., 2002) and mycolic acids from mycobacteria (Barboni et al., 2005), but in contrast to the oligomerization of Gal-3 after binding to LPS of *E. coli*, binding to mycolic acids inhibited oligomerization of Gal-3 (Barboni et al., 2005). Gal-3 binds phosphatidylinositol mannosides from mycobacteria and accumulates in the phagosomes that contain live mycobacteria (Beatty et al., 2002). In association with Dectin-1 Gal-3 also binds the oligomannans on the cell wall of certain pathogenic fungi, and this interaction is associated with enhanced production of TNF-*α* in macrophages in response to either *Saccharomyces cerevisiae* or the pathogenic *C. albicans* (Esteban et al., 2011). Further, Gal-3 binding to *β*-1,2-linked oligomannans of *C. albicans* directly induces the death of these fungi (Kohatsu et al., 2006). Gal-3 deficient mice are more susceptible to cryptococcosis development than wild type mice, since Gal-3 exerts a direct lytic effect on *Cryptococcus neoformans* extracellular vesicles (Wolf et al., 2014). Recently, it has been shown that Gal-3 affects fungal infections by modulation of antifungal immunity, recruitment of neutrophils to the site of infection in mouse model of lung infection with *Aspergillus fumigatus* (Shevchenko et al., 2018). Gal-3 also binds to protozoans, *Leishmania major* (Sato et al., 2014) and *Trypanosoma cruzi* (Moody et al., 2000). Binding of CRD domain of Gal-3 to three proteins of *Trypanosoma cruzi* favors the attachment of these proozoas to host laminin (Moody et al., 2000). Also, Gal-3 mediates the binding of *T. cruzi* to human coronary artery smooth muscle cells, silencing Gal-3 gene in these cells dramatically reduces infection, which indicates that Gal-3 participates in the process of parasite entry into the cells (Kleshchenko et al., 2004). However, decreased secretion of proinflammatory cytokines in spleen and heart of *Trypanosoma cruzi* infected Gal-3 deficient mice has been shown, with elevated mast cell recruitment and fibrosis of heart tissue, indicating that Gal-3 plays a key role in controlling *Trypanosoma cruzi* infection and in preventing heart damage and fibrosis (Kleshchenko et al., 2004).

Since different microbe induce enhanced expession of Gal-3, there is a strong possibility that its expression is also enhanced in periodontal diseases. Gal-3 expressed during infections, modulates the adhesion of microbes to epithelial surfaces, phagocytosis of ingested microorganisms, activation of ERK, AKT, and JAK/STAT1 signaling pathways in epithelial cells and subsequent release of cytokines from these cells, and functional activities of immune cells (Chen et al., 2005; Quattroni et al., 2012; Nita-Lazar et al., 2015; Subhash et al., 2016; Cockram et al., 2019; Gilson et al., 2019). By these mechanisms of direct and indirect modulation of antimicrobial activities Gal-3 can further affect already altered microbiota immune system homeostasis and possibly development of periodontitis.

### Gal-3 and Inflammatory Response

Although the understanding of microbiota in oral cavity is significantly increased (Abusleme et al., 2013), their effects on shaping the immune reactions in the gingival tissue are still poorly explored. Innate defenses of saliva (antimicrobial peptides histatins, lysozyme, lactoferrin, peroxidases, and immunoglobulins) control the oral microbiota (Ross and Herzberg, 2016; Billings et al., 2017). Human whole saliva contains exosome-like vesicles that contain Gal-3 that by enhancing phagocytic activity of neutrophils and by stimulation of cytokines production, might play a regulatory role in local immune defense in the oral cavity (Ogawa et al., 2008). Human gingival tissue contains innate lymphoid cells and natural killer cells but their roles in the immune homeostasis in disease development still need to be established (Dutzan et al., 2016). The crucial role of T and NKT cells in oral cavity protection from viral infections has been documented in patients with severe combined T/NKT immunodeficiencies (Betts et al., 2015; Moutsopoulos et al., 2015).

The dominant population of immune cells, about 95%, in healthy gingival crevice are neutrophils (Delima and Van Dyke, 2003), with significant increase of their number in inflammatory conditions (Dutzan et al., 2016; Rijkschroeff et al., 2016). Neutrophils continuously extravasate into the gingival tissue and with high flow rate pass into the gingival crevice through junctional epithelium (Schiött and Loe, 1970), but the role of neutrophils in the microbial surveillance by extra- or intra-cellular phagocytosis in oral cavity, or by secretion of antimicrobial peptides, is not completely clear (Amulic et al., 2012; Hajishengallis and Hajishengallis, 2014). However, the studies of the patients with primary deficiencies of neutrophils indicate that these cells play the key role in periodontal immune homestasis. Patients with congenital neutrophil deficiencies always have clinical manifestations of severe or aggressive periodontal diseases accompanied with dysregulated IL-17/Th17 immune response (Amulic et al., 2012; Hajishengallis et al., 2014), (Moutsopoulos et al., 2015), which implicates immunoregulatory role of neutrophils in oral cavity (Kruger et al., 2015). Neutropenia is associated with exacerbated IL-23/IL-17 response and thus become a key factor in immunopathological process of periodontal disease development (Moutsopoulos et al., 2014; Ley, 2017; Moutsopoulos et al., 2017). However, excessive number of neutrophils within the gingiva, can contribute to periodontal immunopathology (Kantarci et al., 2003; Matthews et al., 2007; Eskan et al., 2012; Dutzan et al., 2016).

The role of neutrophils is crucial during acute inflammatory response to microbes. Gal-3 is involved in different processes during the acute inflammatory response to microbes including neutrophil activation and adhesion (Kuwabara and Liu, 1996), chemoattraction of monocytes⁄ macrophages (Sano et al., 2000), opsonization of apoptotic neutrophils (Karlsson et al., 2009), and degranulation of mast cells (Frigeri and Liu, 1992). Gal-3 is also able to bind cell surface integrin and thus promotes the adhesion between neutrophils and vascular endothelial cells and stimulates inflammation induced by microbes (Sato et al., 2002). Further, secreted Gal-3 cross-links surface glycoproteins and stimulates the oxidative burst in neutrophils (Yamaoka et al., 1995).

In murine model of pneumonia it has been shown that Gal-3 accumulates in the alveoli after infection with *Streptococcus pneumoniae* and this accumulation of Gal-3 is in correlation with the onset of neutrophil extravasation, suggesting the role of Gal-3 in neutrophil activation (Sato et al., 2002). Gal-3 is able to bind neutrophils by both C-terminal CRD and the N-terminal nonlectin domain, and to induce their activation (Nieminen et al., 2005). In extravasated neutrophils Gal-3 activates the NADPH oxidase (Karlsson et al., 1998). However this Gal-3 mediated neutrofil extravasation appears to be dependent on the microbe that causes infection, since this was not observed in *Escherichia coli* induced pneumonia (Sato et al., 2002). Direct bacteriostatic effect of Gal-3 on *Streptococcus pneumoniae* that reduces pneumonia and bacteriemia has been also demonstrated in this study (Sato et al., 2002). Gal-3 deficiency has been found to be associated with inadequate host response to pneumococcal infection, resulting in high mortality of the infected Gal-3 knockout mice (Farnworth et al., 2008; Nieminen et al., 2008; Wright et al., 2017), while exogenous Gal-3 enhances phagocytosis of *Streptococcus pneumoniae* by both WT and Gal-3 deficient neutrophils (Nieminen et al., 2005; Farnworth et al., 2008). Similar antimicrobial acivity of Gal-3 has been demonstrated against the fungus *Candida albicans* (Kohatsu et al., 2006). On the other hand negative regulation of neutrophil effector functions by cell intrinsic Gal-3 has been reported (Wu et al., 2017). In a model of mouse infection by *Leishmania major* in mice, Gal-3 deficiency resulted in a decrease in the number of infiltrating neutrophils in the skin and enhanced parasite burdens (Bhaumik et al., 2013). Injection of exogenous Gal-3 induced neutrophil migration to the site of injection, suggesting that Gal-3 acts as a DAMP to induce neutrophil migration (Bhaumik et al., 2013).

Gal-3, by opsonization of apopototic neutrophils and facilitation of their phagocytosis by macrophages, plays important role in removal of neutrophils from the inflammed tissue and thus terminates an inflammatory response (Karlsson et al., 2009). This has been also shown *in vivo* in a model of self-resolving peritonitis, where Gal-3-deficient mice showed increased numbers of neutrophils in the peritoneum due to impaired neutrophil apoptosis, efferocytosis and clearance (Wright et al., 2017). On the other side Gal-3 induces neutrophils infiltration, release of inflammatory cytokines, vascular injury, and release of inflammatory mediators from neutrophils in the mice with lethal infection by *Franciscella novicida* enhancing thus lung tissue damage and mortality in comparison with Gal-3 deficient mice, despite similar bacterial burden (Mishra et al., 2013)*.*

In summary these data indicate possible dual effect of Gal-3 on neutrophil functions and tissue damage induced by inflammatory response to microbes depending on the type of microbes, amount and localization of Gal-3. It can be assumed that Gal-3 in basal conditions contributes to homeostasis maintaining in periodontal tissue, while in the conditions of disturbed homeostasis by activation, recruitment and enhancment of effector functions of neutrophils, it participates in immunopathogensis of periodontal diseases.

Gingiva contains several populations of mononuclear phagocytes, dendritic cells (DCs) (Hovav, 2014), resident macrophages, and recruited (inflammatory) monocytes (Dutzan et al., 2016). By analogy to other barriers, it can be suggested that tissue-resident macrophages in gingiva play the role in the maintanance of mucosal immunity (Grainger et al., 2017), and also in healing and repair, given the high frequency of barrier damage and extreme potential for healing (Sciubba et al., 1978). However, the ontogeny, niche location, all aspects of functionality of gingival resident macrophages are still not clarified. The recruitment of CX3CR1 expressing monocytes into the gingiva in response to bacterial infection (Steinmetz et al., 2016), indicates that the function of inflammatory monocytes in inflammed gingiva is similar to their functions in the other sites. Recruited monocytes in gingiva adopt the phenotype similar to skin-resident Langerhans cells (Capucha et al., 2015). Several populations of dendritic cells reside in the gingiva and are increased during gingival inflammation (Jotwani et al., 2001; Jotwani and Cutler, 2003). These dendritic cells within the epithelium ingest microbial antigenic material and transport it to the lymphoid tissue for presentation to lymphocytes.

Expression of Gal-3 increases during monocytes differentiation into macrophages (Liu et al., 1995) and decreases during differentiation of immature dendritic cells toward mature dendritic cells (Dietz et al., 2000). Gal-3 also augments monocyte–monocyte interactions that lead to polykaryon (multinucleated giant cell) formation, a phenotype associated with alternative macrophage activation (Helming and Gordon, 2007). It is known that Gal-3 stimulates macrophage polarization toward M2 phenotype, since macrophages from Gal-3-deficient mice mainly adopt M1 phenotype (MacKinnon et al., 2008). Gal-3 released by macrophages binds LPS from *Salmonella* spp. and thus attenuates stimulation of macrophages with bacterial LPS and consequently production of proinflammatory cytokines acting thus as a negative regulator of LPS-induced endotoxic shock but, conversely, favors *Salmonella* survival (Li et al., 2008). However proinflammatory effects of Gal-3 on macrophages have been also reported. Microglia secrete Gal-3 which binds TLR-4 expressed by neighboring microglia leading to their classical activation with enhanced production of pro-inflammatory cytokines (Dumic et al., 2006). Gal-3 activates NLRP3 inflammasome, stimulating thus inflammatory phenotype of macrophages and is implicated in the pathogenesis of many chronic inflammatory diseases (Pejnovic et al., 2013; Arsenijevic et al., 2016; Simovic Markovic et al., 2016; Tian et al., 2016; Arsenijevic et al., 2019; Liao et al., 2019; Arsenijevic et al., 2020). Having in mind that tissue-resident macrophages in gingiva play the role in the maintanance of mucosal immunity and healing (Grainger et al., 2017) modulation of their activity by Gal-3 certainly contributes to immunopathogenesis of periodontal diseases. It is possible that Gal-3 in mononuclear cells that reside in gingiva, in response to weak stimuli (Szabo and Petrasek, 2015) induced by repetitive minimal changes of oral microbiota, stimulates NLRP3 inflammasome activation and thus contributes to inflammatory response and further tissue damage.

## The Central Role of Th17 Immunity in Oral Tissue Homeostasis and Periodontitis and The Role of Gal-3 in Th17 Immune Reponse Modulation

The key regulators of gingival tissue homeostasis and immunopathology are Th17 cells (Cheng et al., 2014; Abusleme and Moutsopoulos, 2017; Bunte and Beikler, 2019). Stimulation of epithelial cells with IL-17 is critical for the induction of innate immune response, particularly production of β-defensin 3 (Conti et al., 2009; Conti et al., 2011; Conti et al., 2014; Conti et al., 2016). Examination of animal models and the patients with genetic defects in IL-17 signaling indicate the key role for IL-17 in oral antifungal immunity (Puel et al., 2011; Lionakis et al., 2014). The malfunction or decreased number of Th17 cells in gingival tissue is associated with development of experimental periodontitis (Eskan et al., 2012; Moutsopoulos et al., 2014) and with congenital forms of periodontitis in humans (Moutsopoulos et al., 2014).

Modulation of T helper cells function by Gal-3 could affect the balance between microbiota and the host in the oral cavity and contribute to bacterial overload and initiation of periodontitis. Gal-3 modulates the activation of T lymphocytes (Gilson et al., 2019) and supresses Th17 differentiation induced by agonist of dectin-1 and LPS, molecules that prime dendritic cells for Th17 differentiation (Fermin et al., 2013). Higher Th17 immune response in Gal-3 deficient mice was associated with better protection against fungal infection (Fermin et al., 2013). Since Th17 cells that populate the gingival epithelilal cells, play the key role in immune homeostasis and control of microbiota, Gal-3 deficient mice could be more resistant to periodontitis development due to enhanced differentiation toward Th17 cells in the gingival tissue. However, deficiency of Gal-3 in advanced stages of periodontitis could lead to enhanced alveolar bone destruction mediated by Th17 cells (Dutzan and Abusleme, 2019) and thus have completely opposite effects in comparison with Gal-3 deficiency in conditions of homeostasis or initial disbiosis of oral micriobiom. Recent study has been shown that Gal-3, secreted by tumors, binds directly to glycosylated parts of IFN-γ and IL-12, inducing thus their retaining in the extracellular matrix (Gordon-Alonso et al., 2017) avoiding thus IFN-γ diffusion and the formation of an IFNγ-induced chemokine gradient required for CD8^+^ T cell tumor infiltration. However, this capture of cytokines by extracellular Gal-3 could reduce their binding to specific receptors on target cells inhibiting thus their biological activities. This blockade of IFN-γ and IL-12 in extracellular matrix could shift the balance to a Th2-type response and stabilization of periodontal tissue damage and bone loss (Garlet et al., 2006), which is generally associated with a reduced severity/extent of periodontal tissue destruction (or to a 'stable' condition). Blockade of IL-12 is also associated with attenuation of Th1 immune response and Th1 mediated pathology (Malfait et al., 1998), thus Gal-3 mediated capture of IL-12 could attenuate periodontitis since the role of Th1 cells in initiation of periodontal diseases development is known (Gemmell and Seymour, 2004).

In animal model of chronic inflammatory diseases opposite effects of Gal-3 on disease course have been reported. We have previously shown, in mouse model for primary biliary cholangitis (PBC) induced by infection with *Novosphingobium aromaticivorans*, that Gal-3 contributes to enhanced activation of NLRP3 inflammasome in the liver, higher production of IL-1β and enhanced Th17 immune response that contributes to greater liver tissue damage (Arsenijevic et al., 2019). Gal-3 seems to be necessery for immune response to bacteria, and thus contributes to induction of specific autoimunne response and Th17 mediated damage of small bile ducts. Specific inhibition of Gal-3 during PBC induction with bacteria significantly reduced immune response of the host and almost completely reduced the disease (Arsenijevic et al., 2019). Similar effect of Gal-3 has been reported in dnTGF-βRII mice that spontaneously develop PBC (Tian et al., 2016). Gal-3 enhances NLRP3 inflamasome activation induced probably by gut microbiota and Th17 inflammatory cascade in the livers of dnTGF-βRII mice (Tian et al., 2016). The role of Gal-3 in NLRP3 inflamasome activation and promotion of disease has been also shown in DSS-induced colitis (Simovic Markovic et al., 2016). Attenuated Th17 immune response and tissue damage in Gal-3 deficient mice has been also repoprted in mouse model of rheumatoid arthritis (Forsman et al., 2011) and Concanavalin A induced acute inflammatory liver damage (Volarevic et al., 2012). However, in PBC model induced by strong stimulation of immune system with antigen and adjuvant (Arsenijevic et al., 2016), experimental autoimmune encephalomyelitis induced by immunization with MOG_35–55_ peptide (Jiang et al., 2009), multiple low dose streptozotocin-induced diabetes in mice (Mensah-Brown et al., 2009), acute kidney injury induced by cisplatin (Volarevic et al., 2019), and murine cytomegalovirus induced hepatitis (Stojanovic et al., 2019) Gal-3 plays the protective role, attenuates Th1 and Th17 immune responses and tissue damage. In summary, Gal-3 may have double role in autoimmune processes, depending on the dominant pathogenic mechanisms involved (Radosavljevic et al., 2012) and also could have the opposite effects on the outcome of the same disease, depending on the stage of disease which is most affected.

**FIGURE 2:**
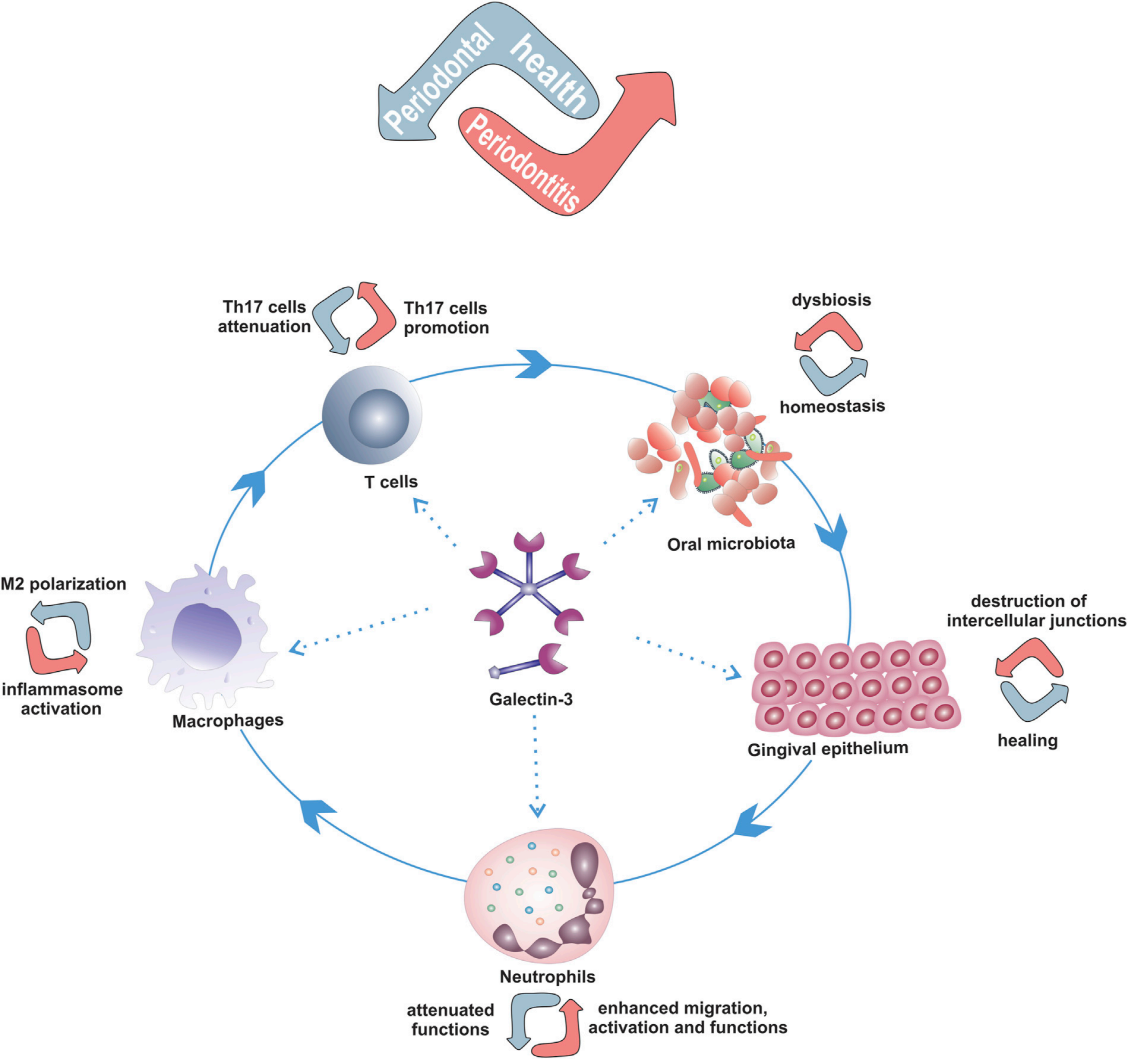
Possible role of Gal-3 in development of periodontal diseases by modulation of the key players in periodontitis pathogenesis. Dual roles of Gal-3 in modulation of oral epithelium, neutrophils, macrophages and Th17 cells functions that depend on amount, extra- or intra-cellular localization of Gal-3 and microenvironement that can contribute to maintenance of periodontal health or periodontitis development.

Given the influence of Gal-3 on microorganisms and immune response, and the findings from bacteria induced PBC and DSS induced colitis, models od diseases where commensal bacteria and normal intestinal microflora play the main role in induction of immune response and disease development, it is conceivable that Gal-3 may have a role in the pathogenesis of periodontal diseases (Figure 2).

## Therapeutic Effects of Gal-3 Inhibition

According to the report that pharmacological inhibition of Gal-3 in adenocarcinomic human alveolar basal epithelial cells significantly attenuates production of IL-17 and suggested pivotal role of Gal-3 in IL-17 mediated inflammation during experimental allergic asthma (Mammen et al., 2018), it can be assumed that Gal-3 deficiency could be protective during periodontal disease development. Protective effects of therapeutic Gal-3 inhibition in several diseases have been reported. Small Gal-3 inhibitor, TD139, enhances production of reactive oxigen species in human neutrophils and ability of mice to clear the fungus, suggesting Gal-3 inhibition as a promising therapeutic strategy for treating systemic candidiasis (Wu et al., 2017). TD139 pretreatment of mice with ConA induced hepatitis attenuates liver injury and infiltration with Th1 and Th17 cells, while enhances infiltration with IL-10 producing T cells (Volarevic et al., 2012). We have also shown that another Gal-3 inhibitor, GM-CT-01, attenuates liver damage in mouse model of bacteria induced PBC (Arsenijevic et al., 2019). Treatment with GM-CT-01 during induction of the diesease with *Novosphingobium aromaticivorans* significantly attenuates liver infiltration with pro-inflammatory macrophages, dendritic cells, NK, NKT, and T cells and reduces expression of NLRP3 inflammasome in the liver infiltrates and interleukin-1β (IL-1β) production in the livers early after infection with of *N. aromaticivorans* (Arsenijevic et al., 2019). It appears that inhibition of Gal-3 during induction phase of PBC just after application of bacteria significantly attenuates immune response to gut commensal and thus almost protect the mice from autoimmune disease induction. GM-CT-01 also significantly attenuates DSS-induced colitis (Simovic Markovic et al., 2016). A novel and orally active Gal-3 antagonist, GB1107, inhibits lung adenocarcinoma growth and metastasis (Vuong et al., 2019). GB1107 exhibits strong immunomodulatory effects, it potentiates the effects of a PD-L1 immune checkpoint inhibitor to increase expression of cytotoxic T cells in tumor tissue (Vuong et al., 2019). Therapeutic effects of Gal-3 inhibition in bacteria induced PBC and DSS induced colitis where intestinal microbiota contribute to disease pathogenesis indicate possible positive effects of Gal-3 inhibition in periodontitis.

Recently, Gal-3 binding core-shell glyconanoparticles based on citrus pectin, with Gal-3 inhibition properties, have been designed for targeted and combination drug delivery (Balakrishnan et al., 2020). These nanoparticles *in vivo* preferentialy accumulate in the Gal-3-expressing tissues of the gastrointestinal tract of mice suggesting their potential for target-specific release of drug used in combination with glyconanoparticles based on citrus pectin and for specific Gal-3 targeting and inhibition of Gal-3-mediated processes (Balakrishnan et al., 2020).

## Conclusion

Since the host-specific inflammatory/immune response to microbes in dental plaques is considered a key element in the immunopathogenesis of periodontal diseases, uncontrolled inflammation, without tendency of resolution, significantly affect the outcome of the process. Gal-3 is the molecule that could affect the functions of the immune and epithelial cells included in the regulation of the inflammation, bacterial clearence and healing. Future studies are needed to dissect the precise role of Gal-3 in maintaining the balance between oral microbiota and the host inflammatory/immune response and periodontal diseases pathogenesis and, considering previously shown therapeutic effects of its inhibition in chronic inflammatory diseases and even possibility of targeted delivery, to point out the Gal-3 as a possible therapeutic target in periodontal disease.
